# Exploring the Association Between Median Arcuate Ligament Syndrome and Hypermobile Ehlers-Danlos Syndrome: A Case Report

**DOI:** 10.7759/cureus.90988

**Published:** 2025-08-25

**Authors:** Kalin R Sorenson, Tanner Buckway, Brandon Hall

**Affiliations:** 1 Surgery, Rocky Vista University College of Osteopathic Medicine, Ivins, USA; 2 Anatomy, Rocky Vista University College of Osteopathic Medicine, Ivins, USA; 3 Clinical Sciences, Rocky Vista University College of Osteopathic Medicine, Ivins, USA

**Keywords:** celiac artery compression syndrome, dunbar syndrome, heds, hypermobile ehler-danlos syndrome, mals, median arcuate ligament syndrome, vascular compression syndrome

## Abstract

Median arcuate ligament syndrome (MALS) is a rare vascular compression disorder resulting from external compression of the celiac artery and surrounding neural plexus by the median arcuate ligament of the diaphragm. It often presents with non-specific symptoms such as postprandial abdominal pain, weight loss, and gastrointestinal distress, making diagnosis challenging. Recent literature suggests a potential association between MALS and connective tissue disorders, particularly hypermobile Ehlers-Danlos syndrome (hEDS), due to increased ligamentous laxity and vascular vulnerability. We present the case of a young adult female with a confirmed diagnosis of hEDS who developed MALS, characterized by chronic epigastric pain worsened by meals, significant weight loss, and an extensive negative gastrointestinal workup. Initial imaging was inconclusive, but subsequent evaluation by a vascular surgeon revealed a low-riding median arcuate ligament with the characteristic “hook-sign” deformity of the celiac artery. A diagnostic celiac plexus block provided temporary relief, supporting the diagnosis, and was followed by robotic-assisted ligament release with bilateral celiac ganglionectomy. Postoperatively, the patient reported substantial improvement in symptoms, though partial recurrence occurred months later, raising concern for post-surgical fibrosis or recurrent vascular compression. This case highlights the diagnostic complexity of MALS, particularly when it overlaps with connective tissue disorders such as hEDS. It emphasizes the utility of celiac plexus blocks as both a diagnostic and prognostic tool and underscores the need for high clinical suspicion in patients with hEDS presenting with chronic, unexplained abdominal pain. Furthermore, it reflects the psychological burden of delayed diagnosis and the impact of receiving definitive treatment. This case is unique in describing the co-occurrence of MALS and hEDS with the associated treatment difficulties, illustrating the complex interplay of vascular compression and connective tissue laxity. It contributes to a limited body of literature exploring how overlapping syndromes complicate diagnosis, surgical decision-making, and long-term outcomes. This case adds to the growing body of evidence linking connective tissue disorders to vascular compression syndromes and supports the need for further research to better define this relationship and improve long-term management strategies.

## Introduction

The median arcuate ligament is a fibrous arch that unites the diaphragmatic crura on either side of the hiatus, usually passing above the origin of the celiac artery [1]. Median arcuate ligament syndrome (MALS), also known as celiac artery compression syndrome and Dunbar syndrome, is a rare condition caused by the compression of the celiac artery and surrounding nerves by the median arcuate ligament, a fibrous band of the diaphragm. The celiac artery provides blood flow to the foregut. Obstruction of this artery disrupts blood flow and can trigger a range of symptoms, such as postprandial abdominal pain, nausea, and unintentional weight loss due to reduced blood flow to abdominal organs [2]. Not all patients present with gastrointestinal (GI) symptoms; this can lead to delayed diagnosis and physician/patient frustration. One study showed 43% of patients did not have GI symptoms, but patients who had GI symptoms were shown to have a higher incidence of joint hypermobility and orthostatic intolerance symptoms (dizziness, syncope, and postural orthostatic tachycardia syndrome) [3]. MALS is more commonly observed in young females between the ages of 30 and 50 years in a 4:1 female-to-male ratio [4].

While the etiology of MALS remains debated, connective tissue disorders may contribute to its pathogenesis. An association between MALS and Ehlers-Danlos syndrome (EDS) is an emerging area of interest, as it predisposes individuals to vascular and ligamentous abnormalities. EDS is a group of inherited connective tissue disorders characterized by joint hypermobility, skin hyperextensibility, and tissue fragility, resulting from collagen abnormalities. These structural defects can increase susceptibility to vascular and anatomical anomalies, including arterial and neural compressions. Because of the abnormal collagen synthesis affecting blood vessels specifically, EDS has been linked to other vascular compression syndromes (VCS), including nutcracker syndrome, superior mesenteric artery (SMA) syndrome, and May-Thurner syndrome [5].

Recent literature suggests that patients with EDS may have an increased risk of MALS and other VCS due to inherent connective tissue weaknesses that compromise vascular stability [6]. Additionally, the shared symptoms of abdominal pain, postprandial fullness, constipation, and diarrhea in both conditions can complicate diagnosis, making it challenging to differentiate MALS from other common hypermobile EDS (hEDS) manifestations [7]. 

Recognizing this association is crucial for appropriate diagnostic workup and management, as EDS patients with MALS may require specialized surgical or interventional approaches that address both vascular compression and underlying connective tissue fragility. Currently, MALS is considered a diagnosis of exclusion, but different imaging modalities, including Doppler ultrasound, computed tomography, magnetic resonance imaging, and mesenteric angiogram, are helpful in diagnosis. Of note, traction has been gained in associating a J-shaped “hook-sign” configuration of the post-stenotic dilation of the celiac artery [1,8-9]. On Doppler ultrasound, an elevated celiac artery peak systolic velocity greater than 200 cm/s and deflection angle greater than 50 degrees are considered supportive for the diagnosis of MALS [4]. 

Treatment is focused on decompressing the celiac trunk, usually by either surgical techniques, including laparoscopic or robotic methods, or interventional procedures such as percutaneous transluminal angioplasty and stenting [10]. The exact prevalence and pathophysiology linking EDS and MALS are still being researched. More studies are needed to confirm this association and guide management [6].

The subsequent case contributes a novel perspective by documenting the diagnosis and surgical treatment of MALS in a patient with hEDS and the associated postoperative challenges that can occur. While individual associations between EDS and VCS have been noted, there is limited literature describing the combined diagnostic, surgical, and postoperative challenges when multiple syndromes such as MALS and hEDS are present concurrently. Our case highlights this intersection and underscores the importance of a multidisciplinary approach to care. It also highlights a potential structural mechanism by which connective tissue fragility in hEDS may predispose to both initial compression and recurrence after treatment. By documenting diagnostic and therapeutic challenges in this patient, this case contributes to the growing literature on the overlap between MALS and connective tissue disorders and supports the need for more nuanced evaluation and management strategies in this unique patient population.

## Case presentation

The patient is a 21-year-old white female with a diagnosis of hEDS who initially presented in May 2022 with right upper quadrant (RUQ) abdominal pain worsened by food intake. Her medical history is notable for chronic joint instability, numerous musculoskeletal injuries, and multiple orthopedic surgeries, including bilateral ankle ligament repairs. She has a Beighton score of 4, which falls one point below the threshold for her age; however, she endorses two positive responses to the five-part historical questionnaire recommended in the 2017 international diagnostic Criterion 1b: the ability to place her hands flat on the floor without bending her knees and to bend her thumb to touch her forearm. While she has not undergone genetic testing, her sister has confirmed hEDS via exclusionary genetic testing, a Beighton score of 7, and a similar history of multisystem connective tissue fragility, supporting a clinical diagnosis consistent with 2017 international diagnostic criteria. Neither patient displays features concerning vascular or classical EDS subtypes (e.g., skin fragility, major vascular events, or translucent skin). The patient also endorses a chronic psychosocial burden, reporting physical and emotional exhaustion due to years of chronic pain, functional limitations, delayed diagnoses, and multiple medical interventions. The patient stated, "It is so draining to be in pain all the time with no diagnosis and all doctors turning me away with no solutions, or even any ideas to help lessen the symptoms, just increased that emotional strain. I began to feel hopeless and like I was doomed to live a life of constant pain. This hopelessness led to many depressive episodes where I felt like I was doomed to never feel good again." 

**Figure 1:**
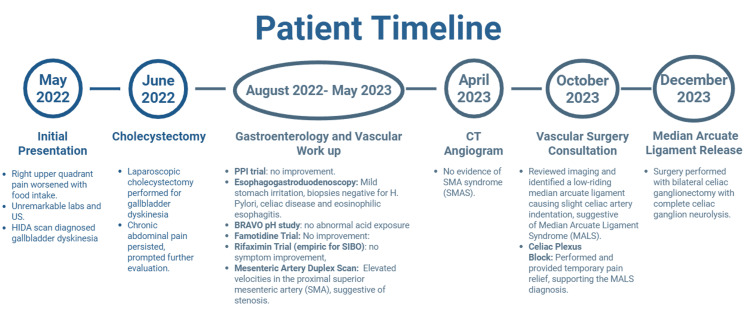
Patient timeline of symptoms, diagnostic workup, and management. Figure created using BioRender.com with a licensed academic account.

Initial evaluation included an abdominal ultrasound and a comprehensive laboratory workup (complete blood count with differential, urinalysis, liver enzymes, and lipase/amylase), all of which were unremarkable. A hepatobiliary iminodiacetic acid scan revealed gallbladder dyskinesia (ejection fraction, 27%), prompting a laparoscopic cholecystectomy in June 2022. Postoperatively, her abdominal pain persisted, prompting further evaluation (Figure 1).

**Figure 2:**
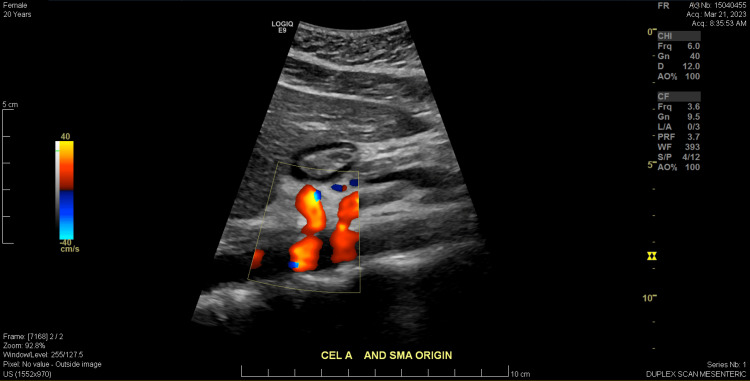
Patient’s abdominal ultrasound capturing the celiac artery and superior mesenteric artery origins. Color Doppler was used to assess for possible stenosis on visual examination, which raised concern for arterial narrowing and prompted further evaluation with flow velocities.

**Figure 3:**
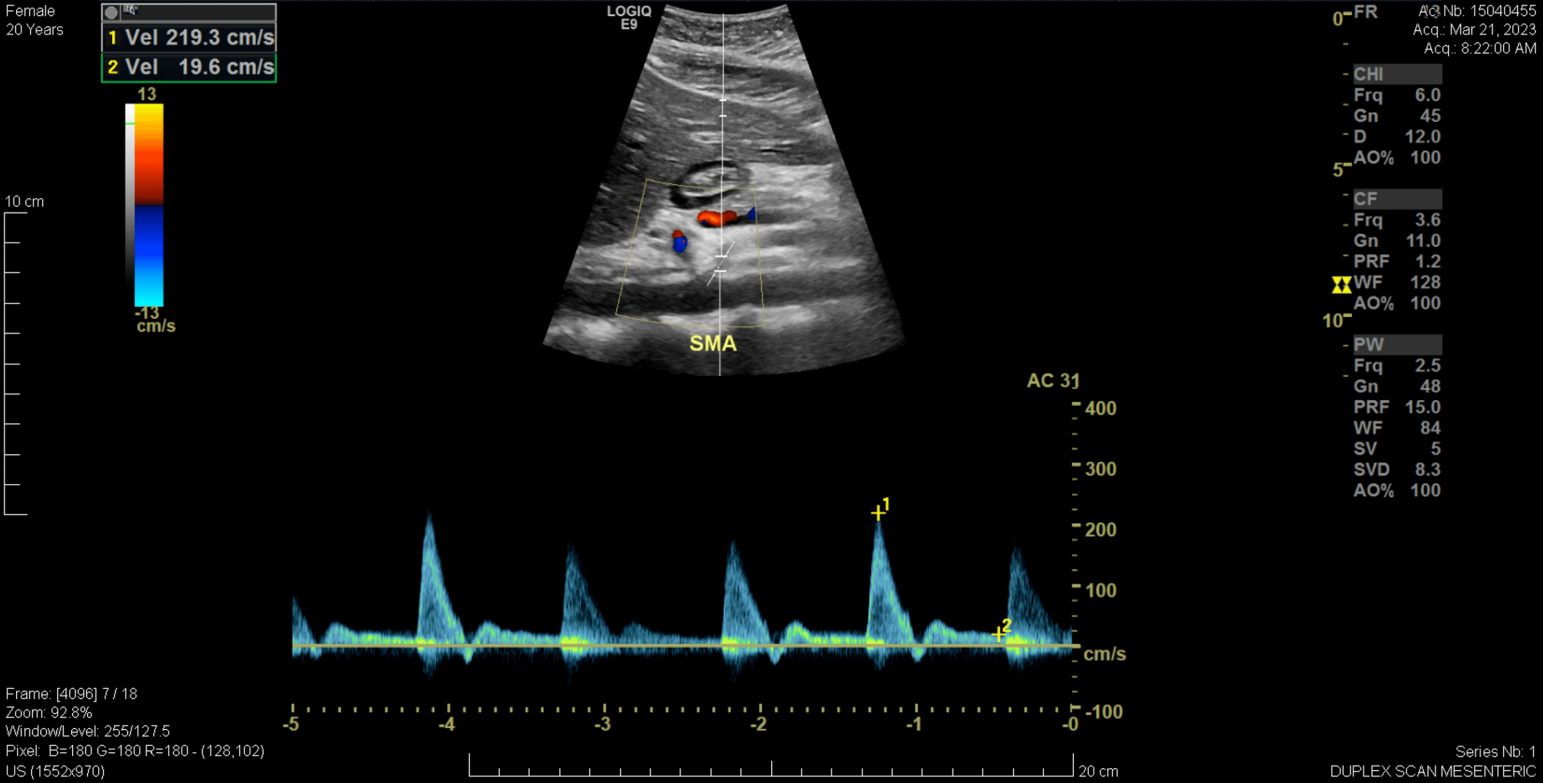
Duplex ultrasound capturing SMA’s peak systolic velocity (219.3 cm/s) at the origin. Elevated velocities were suggestive of superior mesenteric compression. A potential complication of median arcuate ligament syndrome, given the close proximity of the celiac trunk and the low-riding diaphragm, could affect both arteries. SMA: Superior mesenteric artery

Between August 2022 and March 2023, she underwent multiple gastroenterological and vascular assessments, including a proton pump inhibitor (PPI) trial, esophagogastroduodenoscopy (EGD), gastric emptying study, flow cytometry, and mesenteric artery duplex scan. PPI and H2 blocker therapy were ineffective. The EGD showed only mild gastritis, and biopsies were negative for *Helicobacter pylori*, celiac sprue, and eosinophilic esophagitis. Bravo™ pH capsule (Medtronic, Minneapolis, MN, USA), a wireless device used to monitor gastroesophageal reflux, was also used but did not reveal an abnormal amount of acid exposure in the esophagus. A trial of rifaximin was initiated for potential small intestinal bacterial overgrowth; however, no testing was performed due to the facility's lack of testing capabilities. This trial also failed to provide symptom relief. A March 2023 duplex scan (Figures 2, 3) revealed elevated velocities in the proximal SMA, suggestive of SMA stenosis, but a subsequent computed tomography angiogram in April 2023 ruled out SMA syndrome.

**Figure 4:**
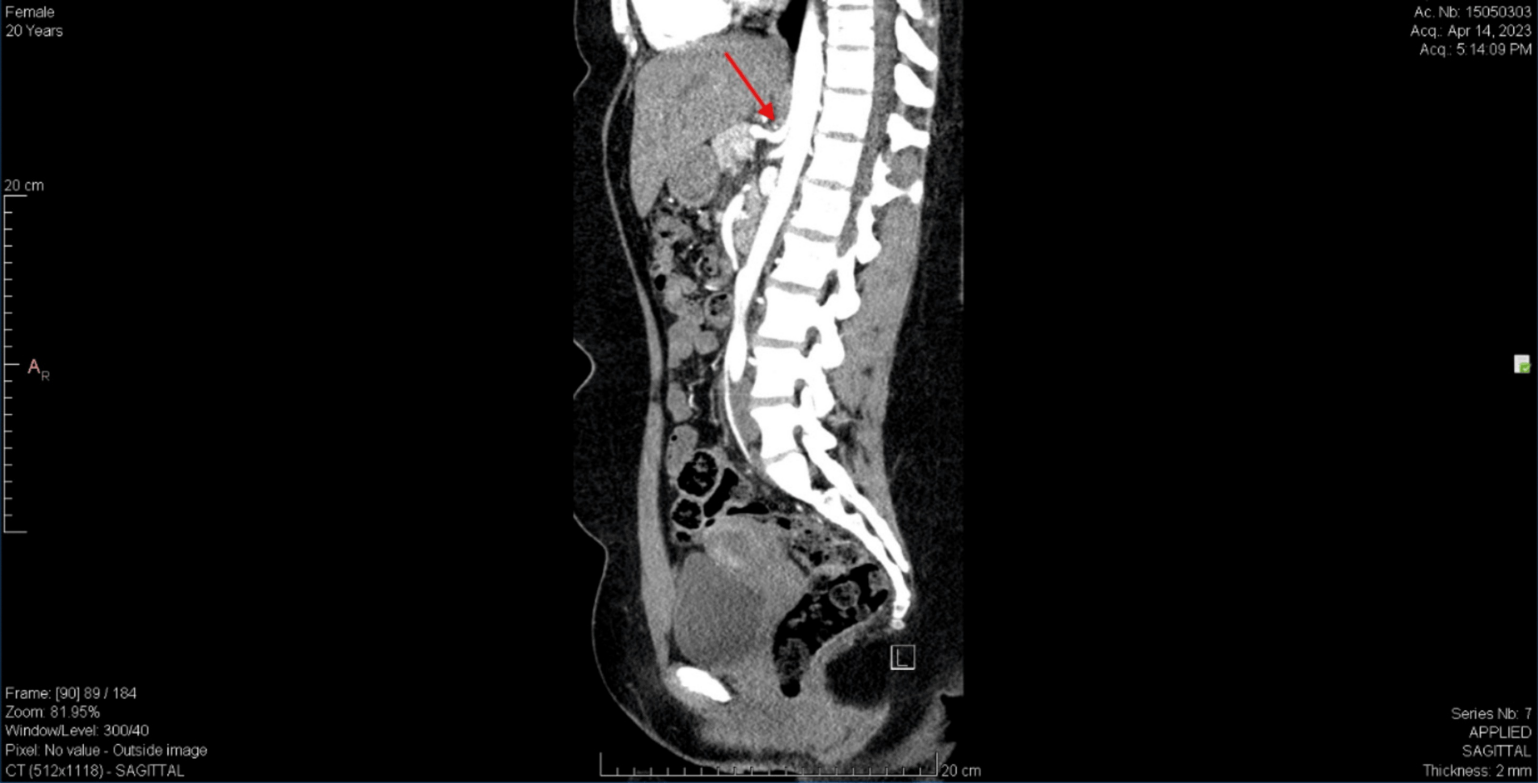
Sagittal CT scan of a patient with a low-riding median arcuate ligament abutting the celiac artery, causing slight vessel indentation, commonly called the “hook sign” due to the artery's acute angle, suggestive of MALS. Red arrow highlights the focal narrowing (or “hook sign”) of the proximal celiac artery consistent with extrinsic compression. CT: Computed tomography; MALS: Median arcuate ligament syndrome

In October 2023, a vascular surgeon reviewed prior imaging and identified a low-riding median arcuate ligament abutting the celiac artery, causing slight vessel indentation commonly referred to as the “hook sign,” suggestive of MALS (Figure 4). On physical examination, the patient had mild epigastric tenderness without rebound, guarding, or palpable masses. Auscultation for an epigastric bruit was not performed, as the diagnosis was strongly supported by characteristic imaging findings. Joint examination revealed signs of generalized hypermobility consistent with a Beighton score of 4. A diagnostic celiac plexus block resulted in temporary symptom relief, further supporting the diagnosis.

Major barriers to diagnosis in this case were the non-specific nature of MALS symptoms, which mimic more prevalent GI disorders, and the lack of awareness among general practitioners and even specialists regarding its potential association with connective tissue disorders such as hEDS. Although a duplex ultrasound in March 2023 showed elevated velocities in the SMA, suggestive of possible vascular compression, a subsequent computed tomography angiogram was interpreted as normal. This highlights a key issue: the sensitivity and specificity of imaging for MALS are highly dependent on the technique used and the expertise of the interpreting clinician. Static imaging alone may miss subtle signs of vascular compression, especially in patients with atypical anatomy or fluctuating symptoms.

It was not until a vascular surgeon reviewed prior imaging that the characteristic "hook sign" of celiac artery indentation was recognized. This delay illustrates a diagnostic pitfall common in MALS: imaging findings may be present but misread or overlooked when clinicians are unfamiliar with the syndrome. The patient’s temporary relief following a diagnostic celiac plexus block further supported the MALS diagnosis, yet this came only after nearly 18 months of inconclusive evaluations, to which the patient became increasingly frustrated and depressed.

Compounding the difficulty was the underlying hEDS diagnosis, which introduces additional complexity due to its association with multiple VCS and generalized connective tissue laxity. In such cases, overlapping symptoms from structural vascular anomalies can cloud the clinical picture, making accurate diagnosis even more elusive. This case underscores the critical importance of specialist referral, appropriate imaging interpretation, and maintaining a broad differential, especially in patients with known connective tissue disorders who present with persistent, unexplained abdominal pain. This case is a good representation of how vital it is that established diagnostic criteria are followed to better identify and diagnose patients with such disorders.

Surgical intervention

In December 2023, she underwent robotic-assisted median arcuate ligament release with bilateral celiac ganglionectomy. Intraoperatively, significant fibrosis and chronic inflammation were observed around the celiac artery, confirming chronic compression. The ligament was dissected and resected, and neurolysis of the celiac plexus was completed. Anatomical variants included a hepatic artery originating from the SMA and the celiac artery dividing into only the splenic and left gastric branches.

Differential diagnoses

The following differential diagnoses (Table 1) include but are not limited to pathologies that were considered in this patient.

**Table 1:** This table provides differential diagnoses considered in this patient and in other patients with similar symptom presentations. *H. pylori*: *Helicobacter pylori*;* *NSAID: Non-steroidal anti-inflammatory drug; RUQ: Right upper quadrant; SMA: Superior mesenteric artery

Category	Condition	Description
Vascular	Superior mesenteric artery syndrome	Compression of the third part of the duodenum between the SMA and the aorta, leading to early satiety, nausea, and vomiting
	Chronic mesenteric ischemia	Atherosclerotic narrowing of the celiac, superior mesenteric, or inferior mesenteric arteries, causing postprandial pain
	Nutcracker syndrome	Compression of the left renal vein between the SMA and the aorta, sometimes causing abdominal pain, hematuria, or left flank pain
	Median arcuate ligament syndrome	Compression of the celiac artery by the median arcuate ligament of the diaphragm, resulting in postprandial pain
Biliary and pancreatic	Sphincter of Oddi dysfunction	Postprandial RUQ pain due to dysfunctional bile flow regulation
	Gallbladder dyskinesia	Impaired gallbladder emptying causing biliary colic symptoms despite a normal ultrasound
	Chronic pancreatitis	Persistent epigastric pain radiating to the back with fat malabsorption and weight loss
Gastrointestinal	Peptic ulcer disease	Gastric or duodenal ulcers from *H. pylori* or NSAIDs, presenting with burning epigastric pain
	Small intestinal bacterial overgrowth	Bloating, diarrhea, and malabsorption from excessive bacterial growth in the small intestine

Outcome and follow-up

By January 2024, the patient experienced substantial symptom improvement, with decreased RUQ pain and improved oral intake. However, by July 2024, she reported recurrence of epigastric pain to preoperative severity, accompanied by a 10-pound unintentional weight loss. Computed tomography imaging in September 2024 demonstrated possible scar tissue formation and postoperative inflammation at the surgical site. In May 2025, she underwent image-guided cryoablation of the local splanchnic nerves to target persistent pain.

## Discussion

MALS, also known as celiac artery compression syndrome or Dunbar syndrome, is characterized by compression of the celiac artery by a low-lying median arcuate ligament, leading to postprandial abdominal pain due to visceral ischemia. Symptoms often include epigastric pain, nausea, emesis, bloating, and diarrhea, similar to the presentation in this case. An epigastric bruit may be heard in up to 83% of patients, often becoming more pronounced during inspiration as the diaphragm moves caudally, further compressing the celiac trunk [11]. Diagnosis is typically one of exclusions, as biliary, pancreatic, and GI causes must first be ruled out. This case highlights the diagnostic complexity of MALS, particularly when initial imaging and assessments fail to reveal the pathology. The patient underwent multiple misdiagnoses before receiving the correct diagnosis, emphasizing the importance of vascular imaging and specialist consultation. Despite initial post-surgical improvement, the patient developed recurrent symptoms, raising concerns for post-surgical fibrosis or vascular complications requiring further monitoring. Additionally, this case suggests a potential association between EDS and MALS, indicating that connective tissue fragility may predispose patients to VCS. The use of a celiac plexus block provided temporary symptom relief, reinforcing the diagnosis of MALS and underscoring the need for a multidisciplinary approach in managing such complex cases. 

The diagnosis of hEDS follows the 2017 international criteria, which emphasize three key components: generalized joint hypermobility (GJH), a constellation of systemic features (including musculoskeletal complications and family history), and exclusion of other heritable and acquired connective tissue disorders [12,13]. Unlike other EDS subtypes, hEDS currently lacks a known genetic marker and remains a clinical diagnosis. Our patient met the necessary criteria through a combination of musculoskeletal history, family history of confirmed hEDS, and exclusion of other connective tissue disorders via history and physical findings. The observed correlation between hEDS and VCS such as MALS has not been well characterized, but underlying connective tissue fragility may contribute to ligamentous laxity or vascular positional anomalies that predispose to compression.

**Figure 5:**
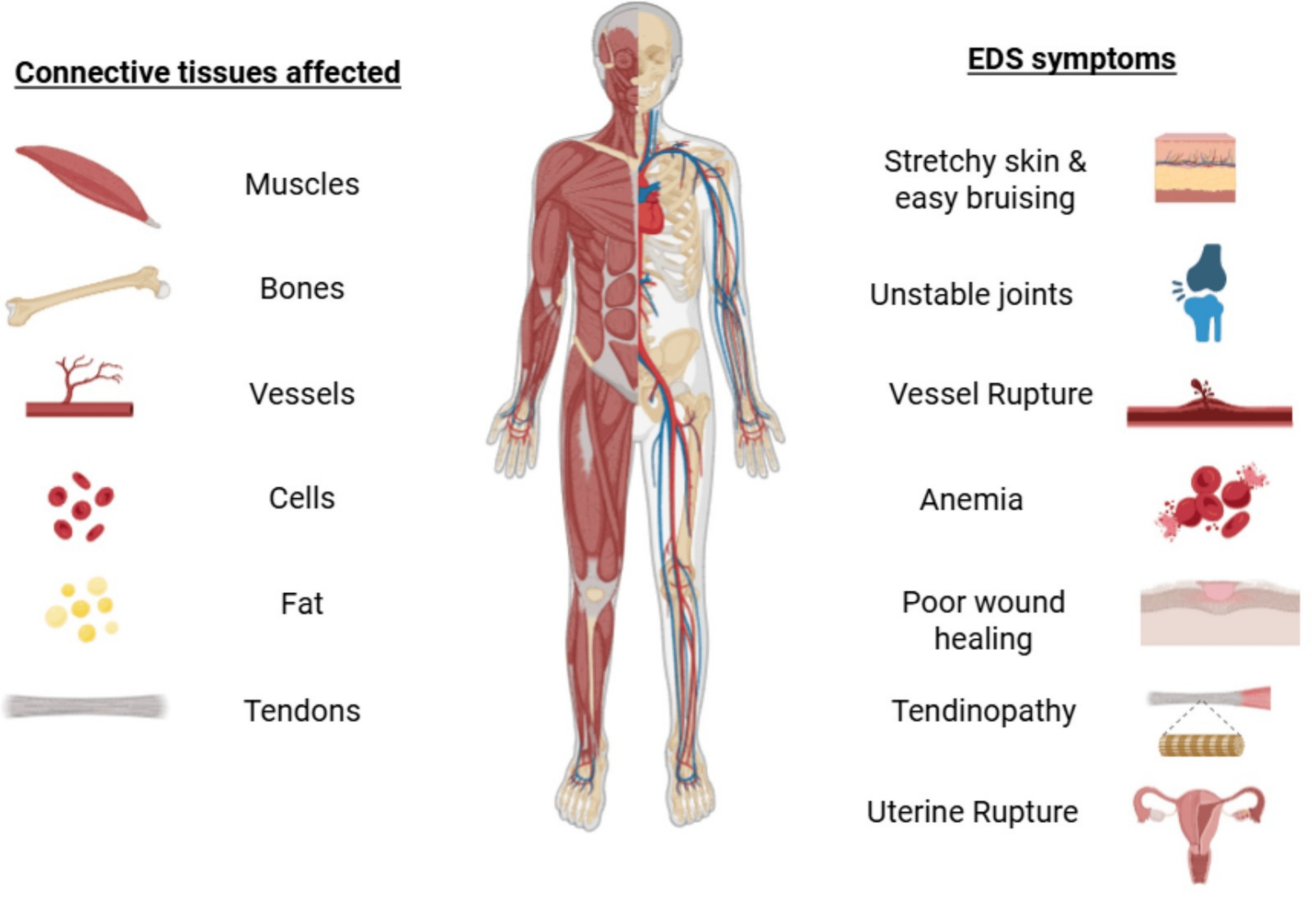
Illustration of potential complications associated with Ehlers-Danlos syndrome (EDS), including vascular, musculoskeletal, and gastrointestinal manifestations. This figure is not specific to the patient in this case but provides context for the multisystem involvement observed in hypermobile EDS and supports the relevance of considering vascular compression syndromes such as MALS in patients with complex symptom presentations. Figure created using BioRender.com with a licensed academic account.

A major strength of this case is the comprehensive clinical history and diagnostic workup, which illustrate the challenges of diagnosing MALS, especially in patients with EDS and symptoms that may complicate diagnosis (Figure 5). The case provides insight into symptom progression, the utility of diagnostic interventions such as celiac plexus blocks, and the temporary relief achieved through surgery. Furthermore, it contributes to the emerging body of research suggesting a link between connective tissue disorders and VCS. The absence of standardized diagnostic criteria for MALS makes it difficult to establish a direct causal relationship between EDS and MALS. Additionally, the recurrence of symptoms following surgical intervention highlights the need for extended follow-up, as long-term outcomes and optimal management strategies remain uncertain. 

This case report has several limitations. One is the incomplete phenotyping of the patient’s hEDS. While the diagnosis was established clinically based on the 2017 international criteria and supported by family history and characteristic features, her Beighton score of 4 falls below the diagnostic threshold for adults per the 2017 criteria. However, per the same guidelines, if a patient scores one point below the threshold, GJH may still be confirmed by a positive response to at least two of five specific historical questions [13]. In this case, the patient reported being able to place her hands flat on the floor without bending her knees and bend her thumb to touch her forearm, satisfying this additional requirement for GJH. Furthermore, genetic testing was not performed to definitively exclude other EDS subtypes, which is an important, though not obligatory, component of the current diagnostic framework when clinical history and physical examination sufficiently support the diagnosis. As with any single-patient report, findings may not be generalizable, and causality between hEDS and MALS cannot be established based on this one case. Additionally, the recurrence of symptoms after surgery introduces uncertainty regarding whether symptom resolution can be reliably sustained through surgical intervention alone. These limitations highlight the need for more rigorous connective tissue disorder evaluation in future studies exploring potential links between hEDS and VCS such as MALS.

Previous studies have reported an increased prevalence of VCS, including MALS, in patients with connective tissue disorders such as EDS, though definitive mechanistic links remain unclear. One study found that patients with hEDS exhibited a higher incidence of MALS and other VCS, aligning with the presentation in this case [6]. Furthermore, other case reports have documented symptom recurrence following surgical decompression, with some suggesting that fibrosis and residual autonomic dysfunction may contribute to persistent or returning symptoms [14]. This case also reinforces findings that celiac plexus blocks can provide both diagnostic and therapeutic benefits in MALS, supporting their role as an adjunct in clinical evaluation and management. 

**Figure 6:**
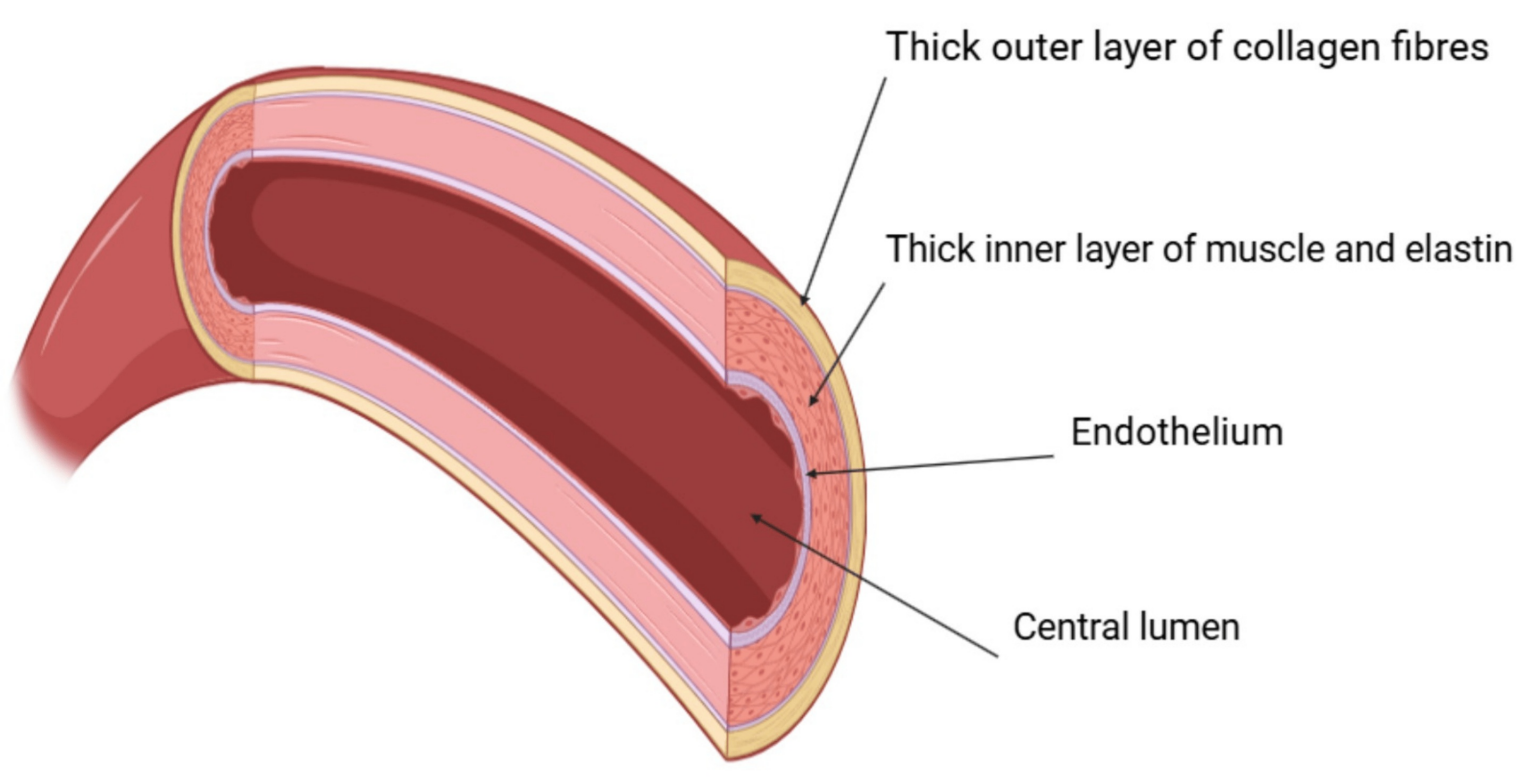
Conceptual diagram of an artery with a thick collagen-rich outer layer, which may be structurally compromised in Ehlers-Danlos syndrome, increasing the likelihood of compression or tearing. While not derived from this patient, the image illustrates a key pathophysiologic feature that may predispose individuals with hEDS to arterial compressions such as those observed in MALS. Figure created using BioRender.com with a licensed academic account. hEDS: Hypermobile Ehlers-Danlos syndrome; MALS: Median arcuate ligament syndrome

In this patient, the underlying hEDS manifested as chronic joint instability, multiple musculoskeletal injuries, and a Beighton score of 4, with additional supportive historical features meeting the 2017 diagnostic criteria. These signs of connective tissue fragility likely contributed to abnormal laxity and elongation of the median arcuate ligament and surrounding structures. Such hypermobility may increase the risk of dynamic vascular compression, particularly during respiration or positional changes, thereby predisposing the patient to MALS. The structural vulnerability of arterial walls in EDS, particularly due to collagen dysfunction in the adventitia, may further increase susceptibility to compression (Figure 6). The initial symptom relief following surgical decompression suggests that vascular and neural impingement was at least temporarily alleviated. However, recurrence of symptoms raises concerns for post-surgical fibrosis, residual autonomic dysfunction, or persistent vascular compromise, mechanisms that may be amplified in patients with underlying connective tissue disorders. The patient’s prolonged diagnostic course and history of misdiagnosis underscore the complexity of MALS in the setting of hEDS and the importance of maintaining a high index of suspicion in patients with unexplained chronic abdominal pain, especially when accompanied by signs of connective tissue fragility. This case also adds to the growing evidence that MALS is not solely a vascular disorder but may involve significant autonomic contributions, particularly in the context of syndromic hypermobility.

Increased awareness of the potential link between EDS and MALS is essential for improving early recognition and preventing misdiagnosis. Future research should aim to clarify the pathophysiological connection between connective tissue disorders and VCS. Elective surgical management of MALS, and other vascular disorders, is associated with good outcomes [15]. However, given the risk of post-surgical symptom recurrence, long-term follow-up with vascular and pain specialists should be a standard practice. Additionally, further studies are necessary to assess the efficacy of various surgical and non-surgical management approaches, particularly in patients with underlying connective tissue fragility. Consideration of autonomic dysfunction in patients with MALS and EDS may be crucial in optimizing treatment strategies and improving post-surgical outcomes. Enhanced diagnostic protocols incorporating vascular imaging and adjunctive testing such as celiac plexus blocks may also aid in more accurate and timely diagnosis. Patients who present with symptoms consistent with MALS but lack a diagnosis of EDS, yet demonstrate clinical features suggestive of EDS, may benefit from genetic testing to further explore a potential association between these conditions.

## Conclusions

This case underscores the importance of considering MALS in patients presenting with chronic postprandial abdominal pain and weight loss, particularly when standard GI and vascular workups are inconclusive. In patients with uncertain diagnoses, a celiac plexus block can serve as both a diagnostic and prognostic tool to support clinical suspicion. The association between MALS and connective tissue disorders such as hEDS further highlights the need for a multidisciplinary approach to diagnosis and management. While surgical intervention can provide meaningful symptomatic relief, long-term follow-up is essential to monitor for potential complications, including fibrosis or recurrent vascular compression. Increased awareness of MALS among healthcare providers is critical to reducing diagnostic delays and improving patient outcomes. Further research is warranted to better understand the pathophysiological link between MALS and connective tissue disorders and to optimize treatment strategies for this complex patient population.
